# Supplementary material for: A hybrid additive manufacturing platform to create bulk and surface composition gradients on scaffolds for tissue regeneration
Source: Nat Commun. 2021 Jan 21;12:500. doi: 10.1038/s41467-020-20865-y (PMC7820014; doi:10.1038/s41467-020-20865-y)
Supplement: Supplementary file 1 — Supplementary Information [file 41467_2020_20865_MOESM1_ESM.pdf]

Supplementary Information: A hybrid additive manufacturing platform to create bulk and surface composition gradients on scaffolds for tissue regeneration

*Ravi Sinha, Maria Cámara-Torres, Paolo Scopece, Emanuele Verga Falzacappa, Alessandro Patelli, Lorenzo Moroni, Carlos Mota**

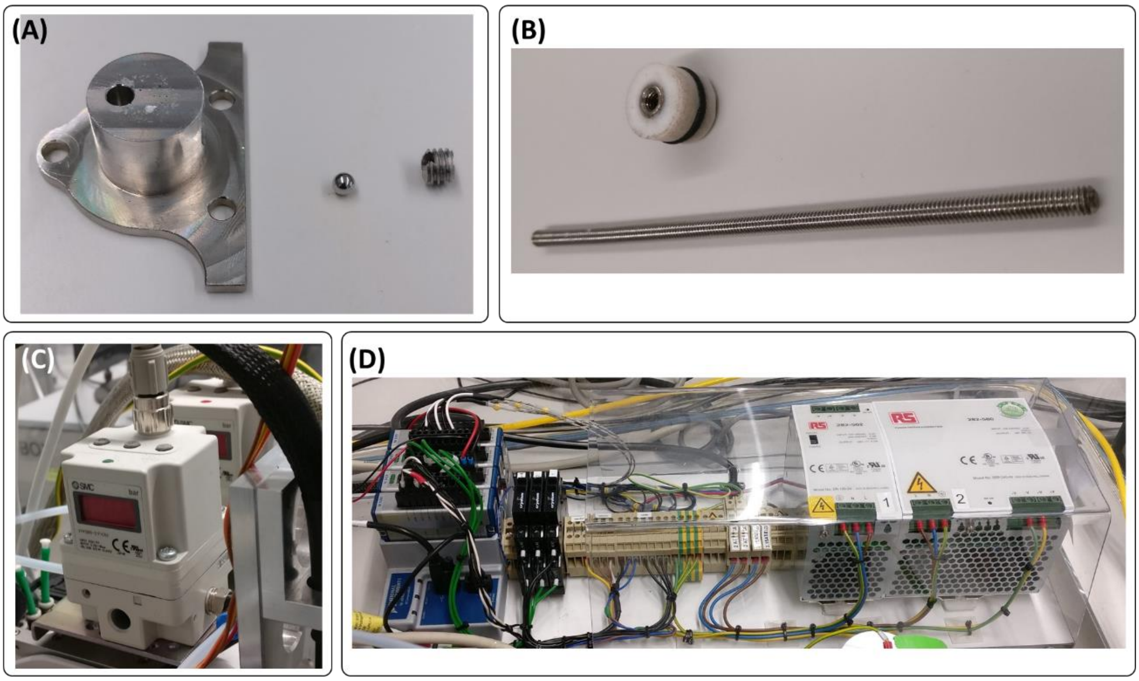

Supplementary Figure 1. Several key supporting technologies enable the proper functioning of the printhead. One-way valves (**A**) at the base of reservoirs prevent back-flows of materials into the other material's reservoir. Extractable plungers (**B**) allow reduction of left-over material in the reservoirs and new material feeding after the reservoirs are emptied. Individual pressure regulators (**C**) and pressure and temperature controllers (**D**) are critical to the printhead operation.

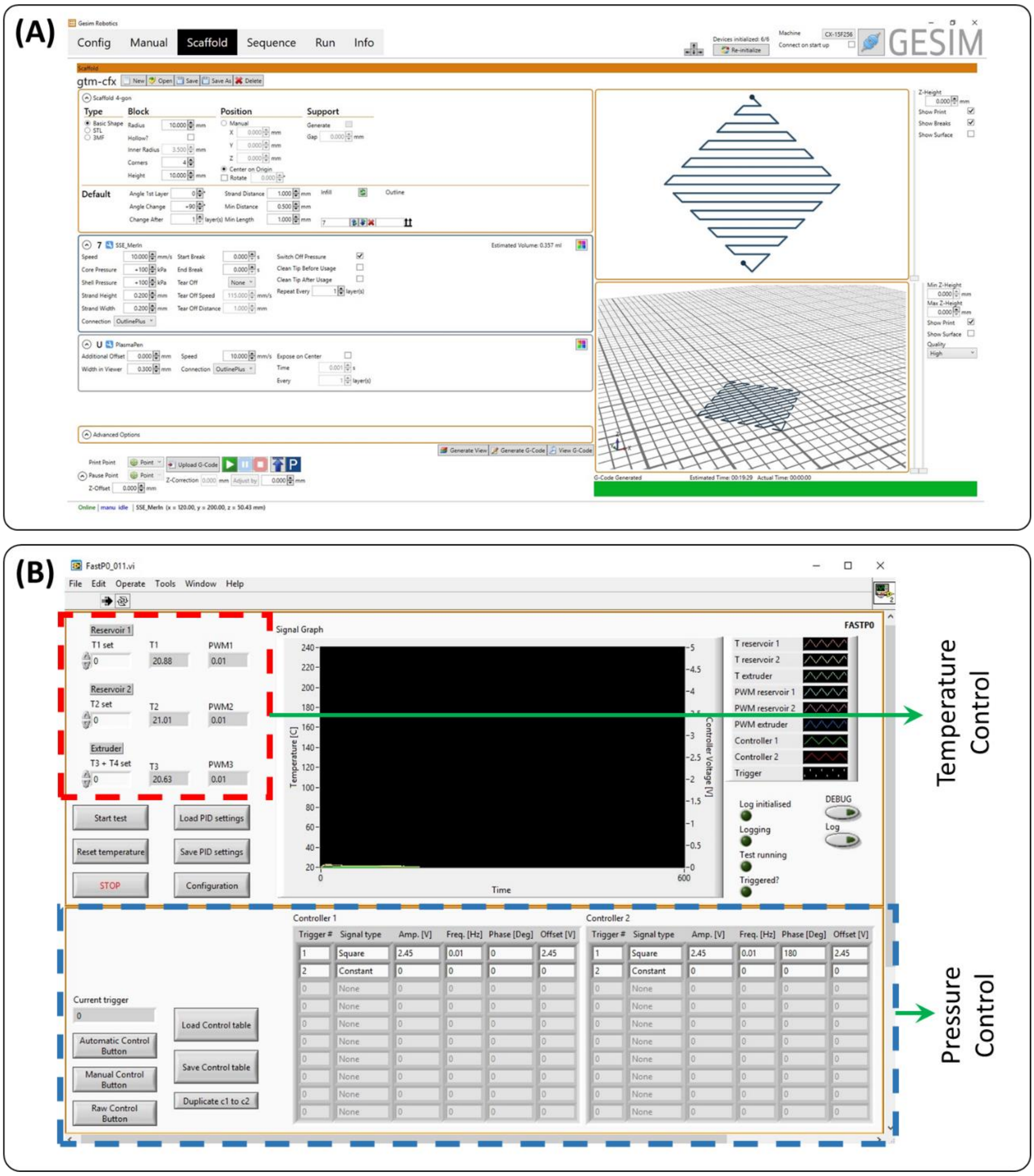

Supplementary Figure 2. Customized software enable the application of the desired print control via a user-friendly interface. The printer software interface (**A**) enables the desired print parameter entry and the automated generation of G-codes to appropriately move and trigger the printhead and the plasma jet. The temperature and pressure software interface (**B**) allows setting the printhead temperatures and the pressure profiles to be applied to each of the printhead reservoirs when triggered.

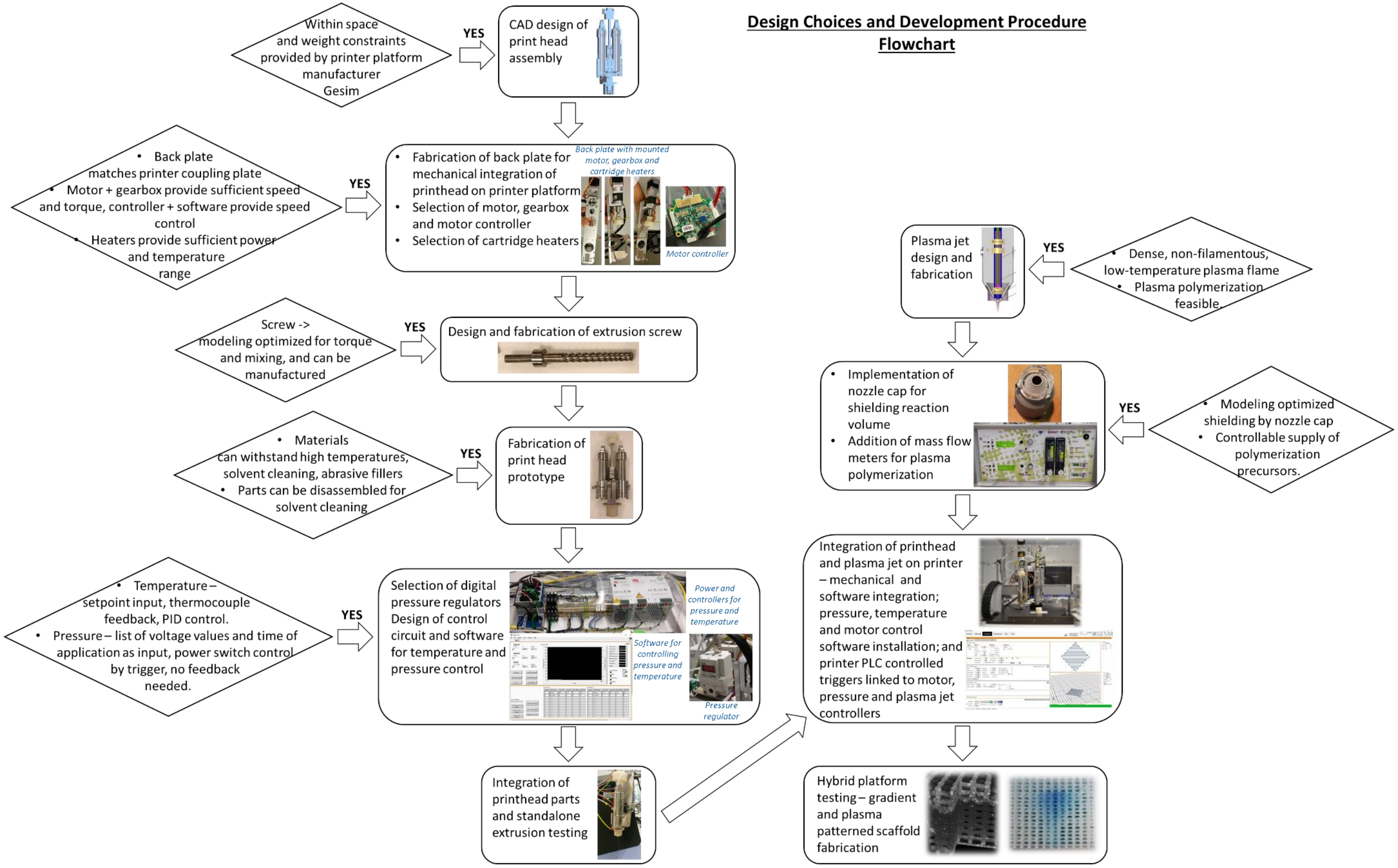
Design Choices and Development Procedure Flowchart
Within space and weight constraints provided by printer platform manufacturer Gesim
YES
CAD design of print head assembly
• Back plate matches printer coupling plate
• Motor + gearbox provide sufficient speed and torque, controller + software provide speed control
• Heaters provide sufficient power and temperature range
YES
• Fabrication of back plate for mechanical integration of printhead on printer platform
• Selection of motor, gearbox and motor controller
• Selection of cartridge heaters
Back plate with mounted motor, gearbox and cartridge heaters
Motor controller
Screw -> modeling optimized for torque and mixing, and can be manufactured
YES
Design and fabrication of extrusion screw
• Materials can withstand high temperatures, solvent cleaning, abrasive fillers
• Parts can be disassembled for solvent cleaning
YES
Fabrication of print head prototype
• Temperature – setpoint input, thermocouple feedback, PID control.
• Pressure – list of voltage values and time of application as input, power switch control by trigger, no feedback needed.
YES
Selection of digital pressure regulators
Design of control circuit and software for temperature and pressure control
Power and controllers for pressure and temperature
Software for controlling pressure and temperature
Pressure regulator
Integration of printhead parts and standalone extrusion testing
• Dense, non-filamentous, low-temperature plasma flame
• Plasma polymerization feasible.
YES
Plasma jet design and fabrication
• Modeling optimized shielding by nozzle cap
• Controllable supply of polymerization precursors.
YES
• Implementation of nozzle cap for shielding reaction volume
• Addition of mass flow meters for plasma polymerization
Integration of printhead and plasma jet on printer – mechanical and software integration; pressure, temperature and motor control software installation; and printer PLC controlled triggers linked to motor, pressure and plasma jet controllers
Hybrid platform testing – gradient and plasma patterned scaffold fabrication

Supplementary Figure 3. Flowchart summarizing design criteria and development procedure for the hybrid AM platform.

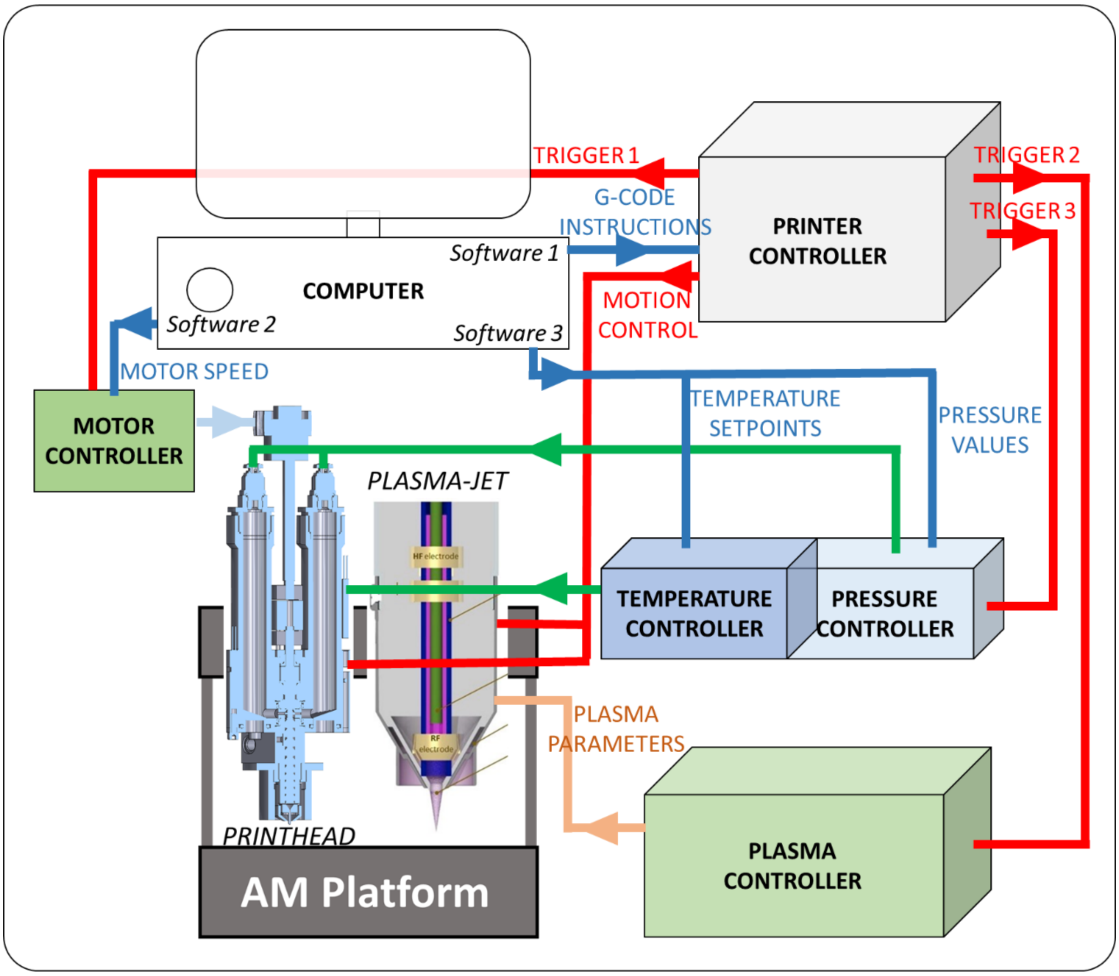

Supplementary Figure 4. The links between the various units and the direction of flow of the control signals are shown schematically for the hybrid AM platform.

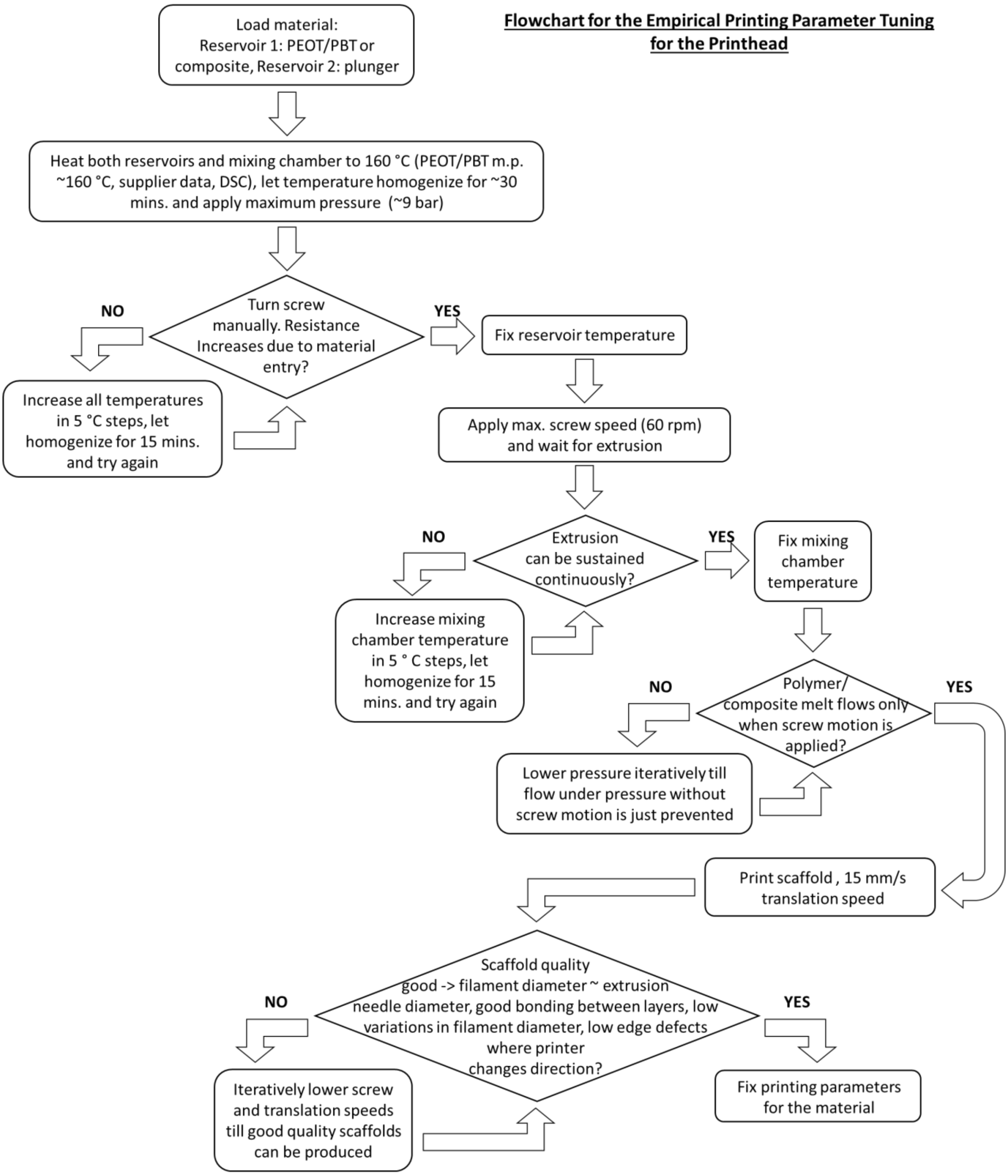

Supplementary Figure 5. Flowchart summarizing how the printing parameters were determined empirically for each material.

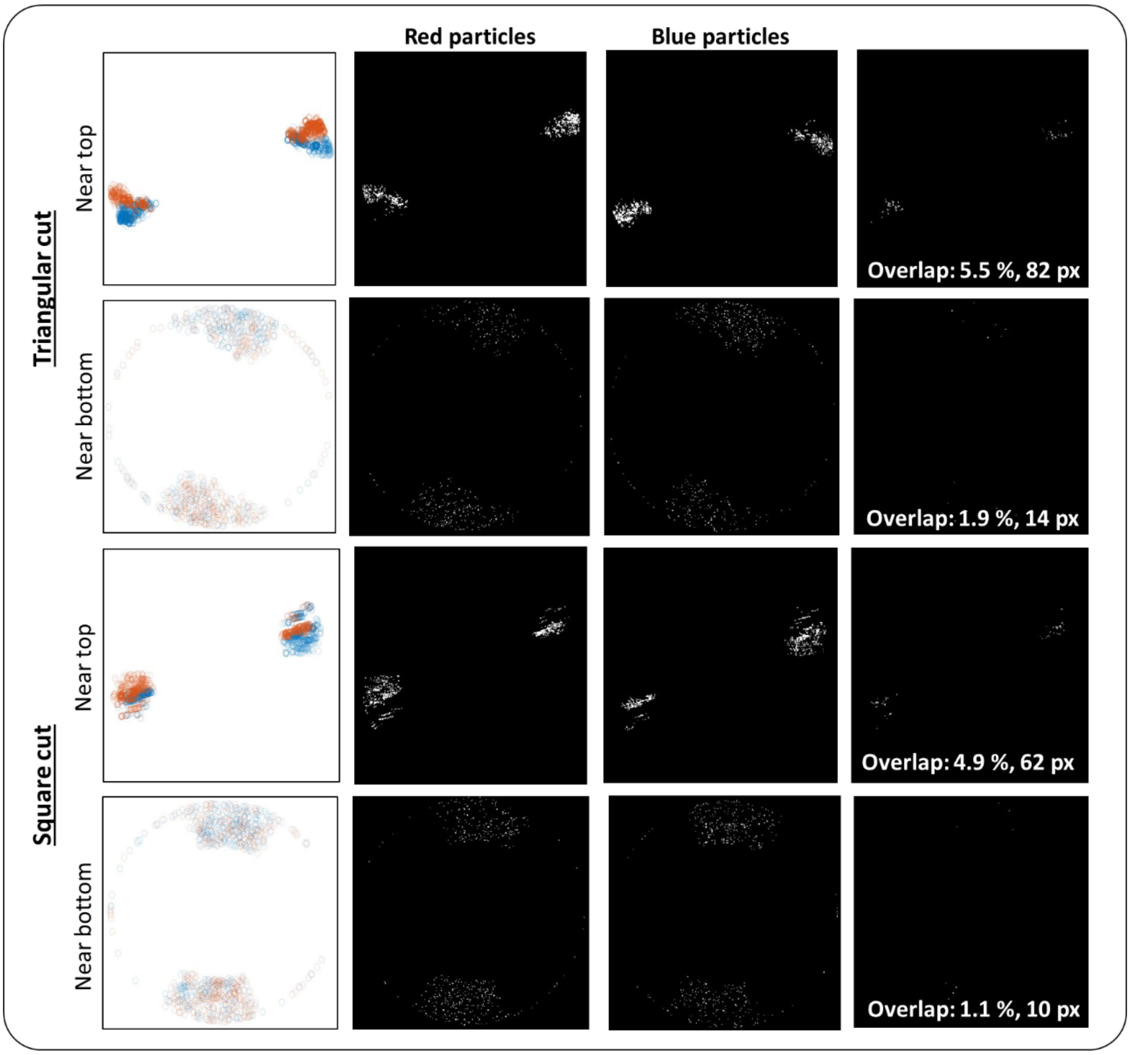

Supplementary Figure 6. Degree of mixing was quantified for particle tracing models with screw rotation. Qualitatively, both designs showed comparable good mixing near the bottom of the modelled screw section, but the overlap metric of mixing did not reflect it, due to the high reduction of particle numbers with increasing distance from the inlets.

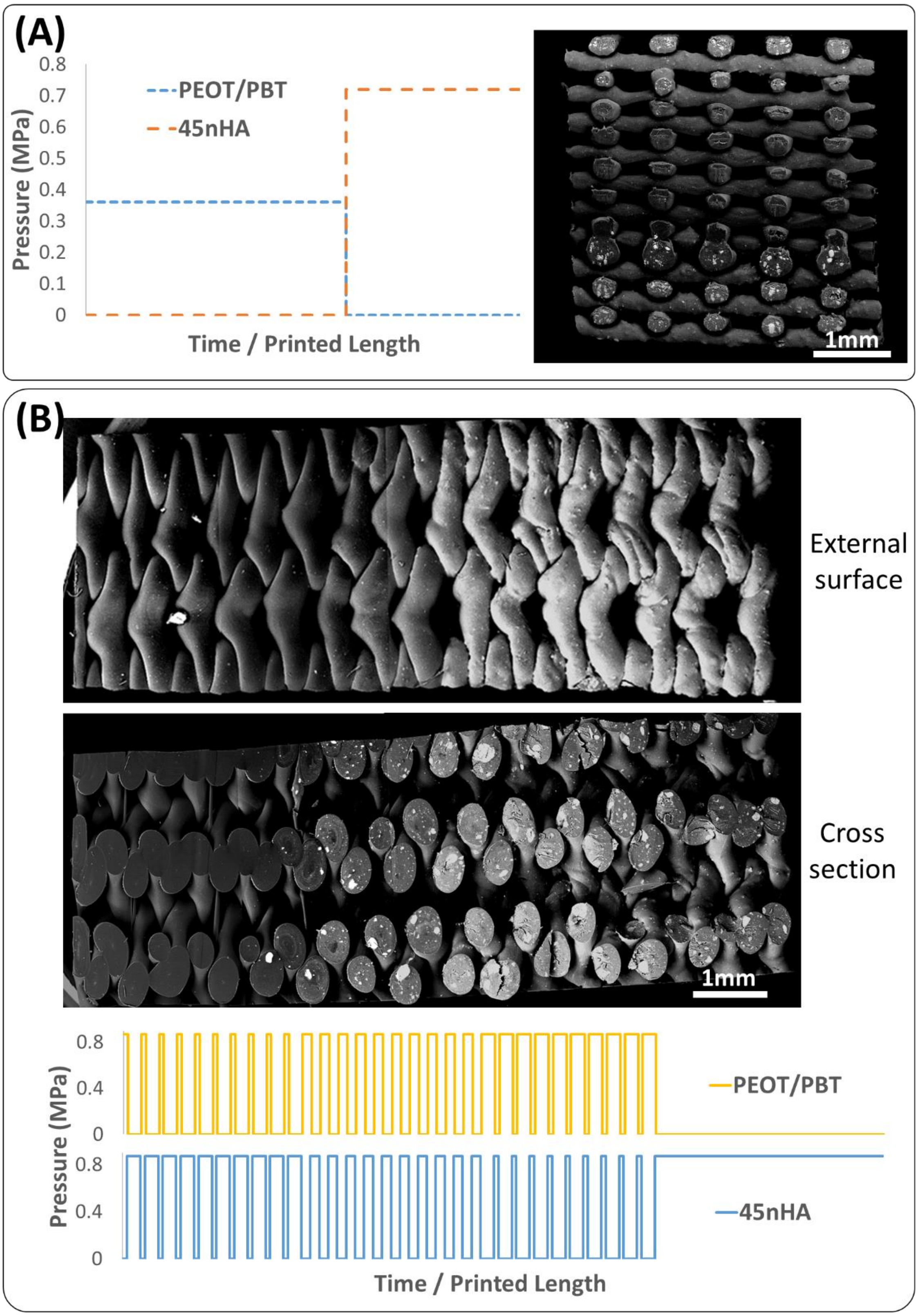

Supplementary Figure 7. Continuous complete composition switches as well as sustained intermediate composition printing were demonstrated. Back-scattered electron-mode scanning electron microscopy (BSE-SEM) images showing HA filler in contrast with the surrounding PEOT/PBT polymer demonstrated that continuous, smooth transitions between compositions could be made when pressures were alternatively applied to the two reservoirs for extended periods of time (**A**). Intermediate compositions could be sustained for a longer

duration by alternatively injecting materials into the screw and controlling their ratio by controlling the ratio of times each pressure is on (**B**).

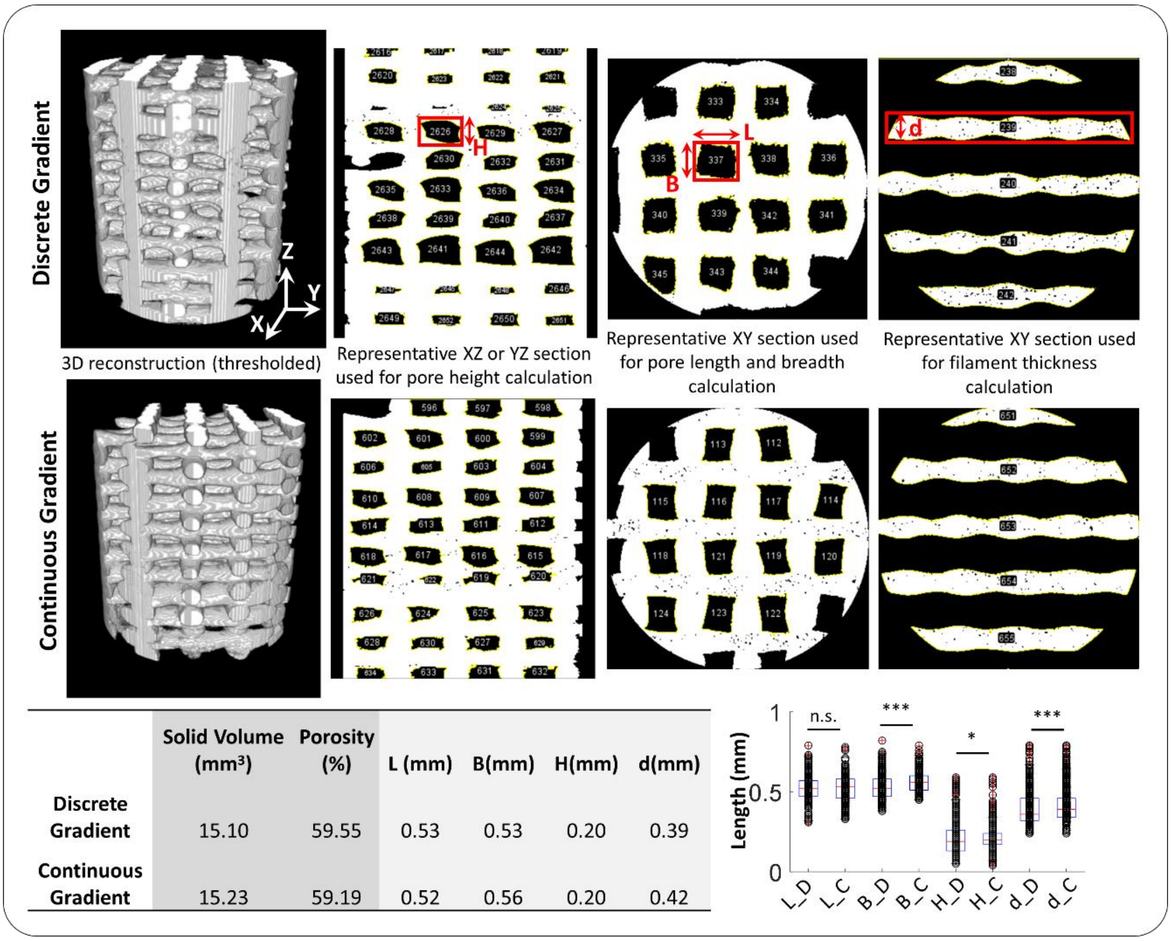

	Solid Volume (mm³)	Porosity (%)	L (mm)	B(mm)	H(mm)	d(mm)
Discrete Gradient	15.10	59.55	0.53	0.53	0.20	0.39
Continuous Gradient	15.23	59.19	0.52	0.56	0.20	0.42

Supplementary Figure 8. Microstructural analysis on representative discrete (D) and continuous (C) gradient scaffolds showed similar average pore sizes (L x B xH), filament diameters (d), and total solid volume and porosity for both scaffolds, as summarized in the table. The differences between the single representative scaffolds in three dimension metrics (B, H, d) were statistically significant (*, $p<0.05$, ***, $p<0.001$) and not significant (n.s.) for L, based on an unpaired t-test, assuming equal variance ($n > 480$ for all metrics). Representative images show which features were measured and plots mark median, upper and lower quartile values as boxes, maxima and minima as whiskers, outliers as red and all individual measurements as black points. Despite the statistical significance of differences, they were very small and not likely to affect the mechanical properties.

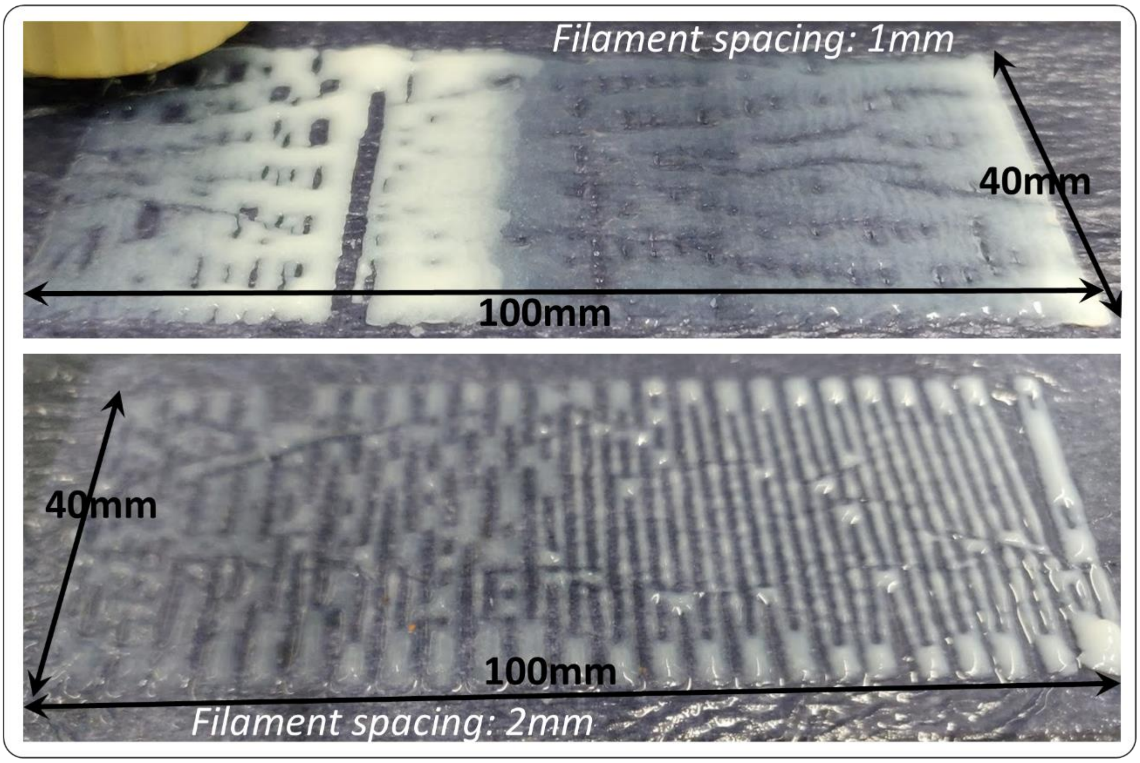

Supplementary Figure 9. Continuous composition gradients can be printed using hydrogels. Alginate gel loaded with hydroxyapatite (HA) was printed on calcium chloride–soaked tissue paper to gel the alginate. Gradients in HA composition could be produced using the printhead.

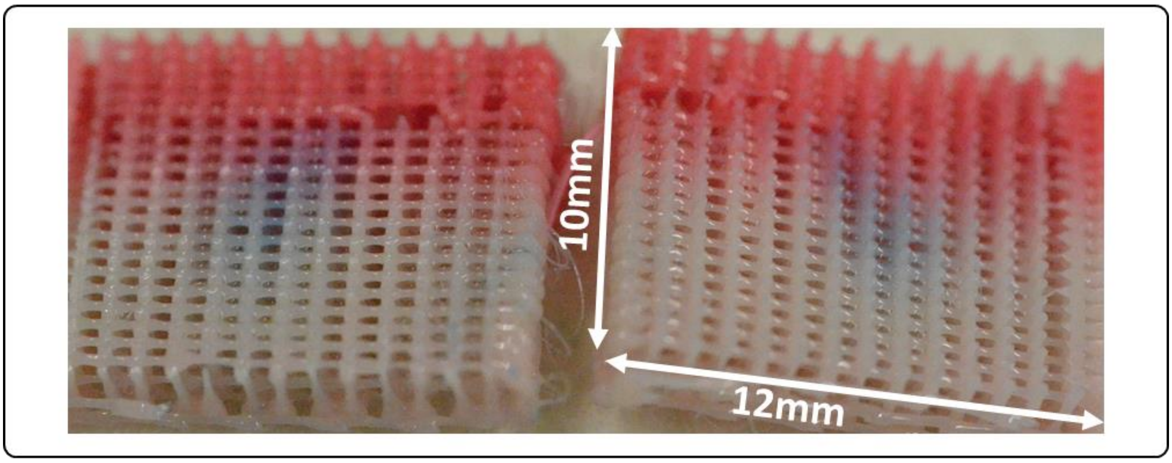

Supplementary Figure 10. Bulk composition gradients can be combined with plasma patterning to simultaneously test for the response of cells to various conditions. Dyed (pink) and non-dyed PEOT/PBT were used to generate the shown composition gradient scaffold, and the core of the scaffold was coated with plasma-polymerized VTMOS-MAA, which is visualized using methylene blue staining.

Supplementary Table 1. Screw geometrical parameters used for the tested designs in the computational modeling–assisted screw operational torque minimization. Column values, in mm, correspond to measurements shown in Figure 2A. Design no. 1 has the base dimensions and for the other designs, the parameter changed is highlighted in bold.

Design No.	t	D1	D2	p1	p2
1	1.5	4.8	5.6	8	6
2	**1**	4.8	5.6	8	6
3	**2**	4.8	5.6	8	6
4	1.5	**4.6**	5.6	8	6
5	1.5	**5**	5.6	8	6
6	1.5	4.8	**5.4**	8	6
7	1.5	4.8	**5.8**	8	6
8	1.5	4.8	5.6	**7**	6
9	1.5	4.8	5.6	**9**	6
10	1.5	4.8	5.6	8	**5**
11	1.5	4.8	5.6	8	**7**

Supplementary Table 2. Printing parameters for various prints and scaffolds produced.

Scaffold type	PEOT/PBT temp. (°C)	PEOT/PBT + filler temp. (°C)	Mixing chamber temp. (°C)	PEOT/PBT pres. profile	PEOT/PBT + filler pres. profile	Screw speed (rpm)	Translation speed (mm/s)	Starting Composition (w/ filler: w/o filler)	Extrusion needle inner diameter (µm)	Strand distance (µm)	Layer height (µm)
**Empirical switching 45nHA**	195	210	220	0.88 MPa, 0.01 Hz sq. wave, phase 0	0.88 MPa, 0.01 Hz sq. wave, phase 180	30	6	50:50	400	1000	320
**Empirical switching 10rGO**	195	220	220	0.45 MPa, 0.002 Hz sq. wave, phase 180	0.88 MPa, 0.002 Hz sq. wave, phase 0	60	3	50:50	250	1000	200
**Empirical switching 20LDH-CFX**	195	185	195	0.88 MPa, 0.01 Hz sq. wave, phase 0	0.88 MPa, 0.01 Hz sq. wave, phase 180	60	10	50:50	400	1000	320
**Empirical switching 20ZrP-GTM**	195	190	195	0.88 MPa, 0.005 Hz sq. wave, phase 0	0.88 MPa, 0.005 Hz sq. wave, phase 180	60	6	50:50	400	1000	320
**Mech. Test – 45nHA**	195	210	220	0	0.72	60	22.5	100:0	250	750	200
**Mech. Test – PEOT/PBT**	195	210	220	0.36	0	60	17.5	0:100	250	750	200
**Mech. Test – HPH_C**	195	210	220	0.36 MPa for layers 1–13	0.72 MPa for layers 14–20	60	17.5 for first 15 layers, 22.5 for next 5 layers	100:0	250	750	200
**Mech. Test – HPH_D**	195	210	220	0.36 MPa for layers 1–5 and 16–20	0.72 MPa for layers 6–15	60	17.5 for first 5 and last 5 layers, 22.5 for middle 10 layers	100:0	250	750	200
**Mech. Test – PHP_C**	195	210	220	0.54 MPa for layers 1–3 and 14–32	0.72 MPa for layers 4–13	20, varied manually a few times	7.5 for layers 15–20, 15 for rest of the layers	0:100	340	850	250
**Mech. Test – PHP_D**	195	210	220	0.54 MPa for layers 1–11 and 22–32	0.72 MPa for layers 12–21	20	15	0:100	340	850	250
