# Supplementary material for: A hybrid additive manufacturing platform to create bulk and surface composition gradients on scaffolds for tissue regeneration
Source: Nat Commun. 2021 Jan 21;12:500. doi: 10.1038/s41467-020-20865-y (PMC7820014; doi:10.1038/s41467-020-20865-y)
Supplement: Supplementary file 2 — Description of Additional Supplementary Files [file 41467_2020_20865_MOESM2_ESM.docx]

**Description of Additional Supplementary Files:**

**Supplementary Movie 1.** Comparison of particle mixing between screws with a triangular and a square thread cut.

**Supplementary Movie 2.** Segmental long bone defect scaffold printing with a cortical composition change.

**Supplementary Movie 3.** Sustained printing of compositions intermediate between the compositions in the two reservoirs.

**Supplementary Movie 4.** Mechanical testing of continuous and discrete gradient scaffolds with a PEOT/PBT region sandwiched between 45nHA regions.

**Supplementary Movie 5.** Mechanical testing of continuous and discrete gradient scaffolds with a 45nHA region sandwiched between PEOT/PBT regions.

**Supplementary Movie 6.** Scaffold production combining a continuous bulk composition gradient and plasma patterning.
