# Supplementary material for: A hybrid additive manufacturing platform to create bulk and surface composition gradients on scaffolds for tissue regeneration
Source: Nat Commun. 2021 Jan 21;12:500. doi: 10.1038/s41467-020-20865-y (PMC7820014; doi:10.1038/s41467-020-20865-y)
Supplement: Supplementary file 9 — Reporting Summary [file 41467_2020_20865_MOESM9_ESM.pdf]

nature research

Corresponding author(s): NCOMMS-20-36701

Last updated by author(s): Dec 9, 2020

Reporting Summary

Nature Research wishes to improve the reproducibility of the work that we publish. This form provides structure for consistency and transparency in reporting. For further information on Nature Research policies, see our Editorial Policies and the Editorial Policy Checklist.

Statistics

For all statistical analyses, confirm that the following items are present in the figure legend, table legend, main text, or Methods section.

n/a	Confirmed	
[ ]	[x]	The exact sample size (*n*) for each experimental group/condition, given as a discrete number and unit of measurement
[ ]	[x]	A statement on whether measurements were taken from distinct samples or whether the same sample was measured repeatedly
[ ]	[x]	The statistical test(s) used AND whether they are one- or two-sided<br>*Only common tests should be described solely by name; describe more complex techniques in the Methods section.*
[x]	[ ]	A description of all covariates tested
[x]	[ ]	A description of any assumptions or corrections, such as tests of normality and adjustment for multiple comparisons
[ ]	[x]	A full description of the statistical parameters including central tendency (e.g. means) or other basic estimates (e.g. regression coefficient) AND variation (e.g. standard deviation) or associated estimates of uncertainty (e.g. confidence intervals)
[ ]	[x]	For null hypothesis testing, the test statistic (e.g. *F*, *t*, *r*) with confidence intervals, effect sizes, degrees of freedom and *P* value noted<br>*Give P values as exact values whenever suitable.*
[x]	[ ]	For Bayesian analysis, information on the choice of priors and Markov chain Monte Carlo settings
[x]	[ ]	For hierarchical and complex designs, identification of the appropriate level for tests and full reporting of outcomes
[x]	[ ]	Estimates of effect sizes (e.g. Cohen's *d*, Pearson's *r*), indicating how they were calculated

Our web collection on statistics for biologists contains articles on many of the points above.

Software and code

Policy information about availability of computer code

Data collection	Not applicable
Data analysis	Not applicable

For manuscripts utilizing custom algorithms or software that are central to the research but not yet described in published literature, software must be made available to editors and reviewers. We strongly encourage code deposition in a community repository (e.g. GitHub). See the Nature Research guidelines for submitting code & software for further information.

Data

Policy information about availability of data

All manuscripts must include a data availability statement. This statement should provide the following information, where applicable:
- Accession codes, unique identifiers, or web links for publicly available datasets
- A list of figures that have associated raw data
- A description of any restrictions on data availability

The source files and other raw data has been uploaded to DataVerse.nl with the DOI https://doi.org/10.34894/A4NGLO, This data availability section is included in the manuscript.

Field-specific reporting

Life sciences study design

All studies must disclose on these points even when the disclosure is negative.

Sample size	Sample size was selected for a minimum of n=3 as scaffolds generally show small variation in mechanical behaviour across samples.
Data exclusions	No data has been excluded from the reported results.
Replication	As reported most experiments were replicable but small adjustments were performed during the scaffold manufacturing, This was dependent to random material aggregation events, sensitivity to changes in ambient conditions, or complex mixture rheological behaviors during dual material processing.
Randomization	Not applicable
Blinding	Not applicable

Reporting for specific materials, systems and methods

We require information from authors about some types of materials, experimental systems and methods used in many studies. Here, indicate whether each material, system or method listed is relevant to your study. If you are not sure if a list item applies to your research, read the appropriate section before selecting a response.

Materials & experimental systems

n/a	Involved in the study	
☒	☐	Antibodies
☒	☐	Eukaryotic cell lines
☒	☐	Palaeontology and archaeology
☒	☐	Animals and other organisms
☐	☒	Human research participants
☒	☐	Clinical data
☒	☐	Dual use research of concern

Methods

n/a	Involved in the study	
☒	☐	ChIP-seq
☒	☐	Flow cytometry
☒	☐	MRI-based neuroimaging

Human research participants

Policy information about studies involving human research participants

Population characteristics	HMSCs (donor d8011L, bone marrow derived, female, age 22) were purchased from Dr. Prockop (Texas A&M Health Science Center, College of Medicine, Institute for Regenerative Medicine) who obtained them with written consent and in accordance with institutional regulations under 45 CF 46.116/ 21 CFR 50.27.
Recruitment	Information available upon request to the provider.
Ethics oversight	Scott & White Institutional Review Board<br>Federalwide Assurance #FWA00003358<br>IRB Registration #IRB00000706

Note that full information on the approval of the study protocol must also be provided in the manuscript.
